# Nonparametric Bayesian clustering to detect bipolar methylated genomic loci

**DOI:** 10.1186/s12859-014-0439-2

**Published:** 2015-01-16

**Authors:** Xiaowei Wu, Ming-an Sun, Hongxiao Zhu, Hehuang Xie

**Affiliations:** 1 Department of Statistics, Virginia Tech, 250 Drillfield Drive, Blacksburg, 24061 VA USA; 2 Virginia Bioinformatics Institute, Virginia Tech, 1015 Life Science Circle, Blacksburg, 24061 VA USA; 3 Department of Biological Sciences, Virginia Tech, 1405 Perry Street, Blacksburg, 24061 VA USA

**Keywords:** DNA methylation, Epigenetics, Nonparametric Bayesian

## Abstract

**Background:**

With recent development in sequencing technology, a large number of genome-wide DNA methylation studies have generated massive amounts of bisulfite sequencing data. The analysis of DNA methylation patterns helps researchers understand epigenetic regulatory mechanisms. Highly variable methylation patterns reflect stochastic fluctuations in DNA methylation, whereas well-structured methylation patterns imply deterministic methylation events. Among these methylation patterns, bipolar patterns are important as they may originate from allele-specific methylation (ASM) or cell-specific methylation (CSM).

**Results:**

Utilizing nonparametric Bayesian clustering followed by hypothesis testing, we have developed a novel statistical approach to identify bipolar methylated genomic regions in bisulfite sequencing data. Simulation studies demonstrate that the proposed method achieves good performance in terms of specificity and sensitivity. We used the method to analyze data from mouse brain and human blood methylomes. The bipolar methylated segments detected are found highly consistent with the differentially methylated regions identified by using purified cell subsets.

**Conclusions:**

Bipolar DNA methylation often indicates epigenetic heterogeneity caused by ASM or CSM. With allele-specific events filtered out or appropriately taken into account, our proposed approach sheds light on the identification of cell-specific genes/pathways under strong epigenetic control in a heterogeneous cell population.

**Electronic supplementary material:**

The online version of this article (doi:10.1186/s12859-014-0439-2) contains supplementary material, which is available to authorized users.

## Background

DNA methylation is a crucial epigenetic modification involved in many biological processes, from normal cellular differentiation to disease genesis and progression. It is part of the epigenetic code recognized as an essential mechanism to stably silence gene transcription and inactivate transposable elements [1]. The gold standard for methylation detection is bisulfite sequencing [2,3]. After the treatment with sodium bisulfite, unmethylated cytosines are converted to uracils and further replaced by thymidines in PCR amplification, while methylated cytosines remain unchanged. The presence of cytosines or thymidines thus indicates the methylation state of a bisulfite treated DNA sequence at the single-base resolution. Since 2008 when the first methylome was determined for *Arabidopsis thaliana*, massive amounts of bisulfite sequencing data have been generated with the rapidly developing high-throughput sequencing technologies. This promotes the advancement of various analytic tools which are mostly devoted to bisulfite sequence processing, mapping [4-7] and methylation profile comparison [8-11].

Most current methylation data sets were derived from tissue samples with heterogeneous cell population. To gain an effective representation, it is usually required to generate multiple sequence reads for each genomic locus. An important feature of bisulfite sequencing data is that each sequence read takes on a methylation pattern, herein defined as the combination of methylation states of neighboring CpG dinucleotides in a DNA strand. A genomic locus can thus be characterized by the methylation patterns presented in multiple sequence reads, which reflect distinct epigenetic control mechanisms. Homogeneous methylation patterns with all sequence reads sharing the same methylation state indicate strong constraints in methylation control for the entire cell population. In contrast, heterogeneous methylation patterns with a high degree of methylation variation suggest stochastic methylation events in the cell population. Recently, we introduced “methylation entropy” [12] as a measurement to assess methylation variation in bisulfite sequencing data. For genomic loci at the same methylation level, the ones with homogenous methylation patterns show lower methylation entropy than those with heterogeneous patterns. We collected data from eight Alu methylomes and used the data to analyze human cerebellum and ependymomas [13,14]. Interestingly, we found that some genomic loci with intermediate methylation level (30%–70% methylated) exhibited significantly low methylation entropy as compared with random simulation [12]. These sequence reads frequently demonstrate bipolar methylation patterns, that is, sequence reads mapped to the same locus exhibiting two distinct methylation patterns: some reads are with heavily methylated CpG sites while other reads are with hypo-methylated CpG sites.

Bipolar methylation patterns are of particular interest as they may originate from allele-specific methylation (ASM) or cell-specific methylation (CSM). ASM has been well recognized in X chromosome inactivation to achieve dosage balance and genomic imprinting, and it may arise during gametogenesis for gametic imprints or in post-implantation embryos for somatic imprints [15,16]. SNPs are the most powerful markers for ASM identification. Recently, a mouse ASM map was generated for the prefrontal cortex tissues derived from reciprocal crosses between two distantly related inbred mouse strains [17]. Over 20 million SNPs present in the genomes of these two strains provide a high density SNP map (one SNP in every 133 bp). A total of 1,952 CG dinucleotides in 55 discrete genomic loci were identified as imprinted. As for the human genome, it was found that the number of imprinted regions are very limited as well [16]. These regions have been well documented in imprinted gene databases such as the catalogue of Imprinted Genes and Parent of Origin Effects [18]. Using the mouse ASM map and the known imprinted gene list as a guide, it is possible to distinguish different sources of partial methylation. Besides the advancement in ASM study, CSM events have also been explored in breadth and depth in many methylation studies to pinpoint the methylation controls underlying cell-fate decisions. Genomic regions associated with CSM have been found in the promoters of lineage-specific genes [19,20], intragenic CpG islands [21], CpG island shores [22] and transcription factor binding sites [23]. Current methods for CSM identification relys on calculating average methylation levels on one or more contiguous CpG sites from purified cell subsets and making inference by pairwise comparison. Despite the effectiveness of these methods, tissues in higher organisms, for instance the brain, often consist of many different types of cells that are difficult to be dissociated. The detection of CSM regions in a heterogeneous cell population still remains a major challenge.

To fill in the research gap, we develop a two-step statistical approach to detect bipolar methylated genomic loci, through nonparametric Bayesian clustering and hypothesis testing. In the clustering step, we adopt a fast Dirichlet-process-mixture (DPM) search method [24] to assign reads to potential hyper/hypo-methylated groups. The subsequent testing step then calibrates the separation of the two groups. Simulation studies demonstrate that our approach achieves good performance in terms of specificity and sensitivity. In addition, the DPM search method is shown to be more accurate in reads assignment than the traditional *k*-means clustering and finite mixture clustering. With ASM loci known and filtered out, our approach can be used to find cell-specific genes/pathways under epigenetic control. Through analyzing recently published data sets on mouse brain and human blood methylomes, we demonstrate that the proposed approach can effectively detect CSM regions. These regions are found highly consistent with the differentially methylated regions (DMRs) identified by using purified neuron-glia cells and neutrophil-B cells.

## Methods

**Figure 1:**
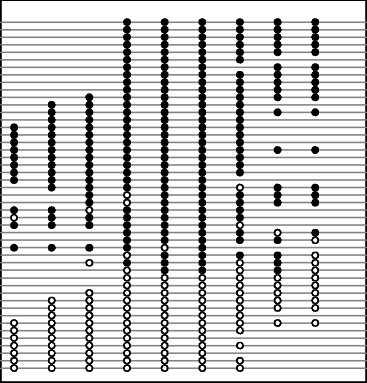
**Promoter methylation pattern of prodynorphin gene in human brain [** 27 **].** This figure shows a genomic region of 95 bp with 9 CpG dinucleotides. Most reads are completely unmethylated (open circles) or methylated (filled circles), but only a few reads are with both unmethylated and methylated cytosines.

In bisulfite sequencing, the methylation state of each CpG site on every mapped read is determined by the presence of cytosines or thymidines. For a particular sequence read, the combination of methylation states on neighboring CpG sites defines a certain methylation pattern, which, for a better understanding, can be thought of as methylcytosine “haplotype” (see Figure 1 for an example). Our purpose is to identify genomic regions with bipolar methylation patterns. To fulfill the purpose, two critical questions need to be answered: 1) How to build a feasible model for bipolar methylation? 2) How to detect bipolar methylation with sufficient statistical power? In what follows, we first introduce a hierarchical model modified from Peng and Ecker (2012) [25] and from Fang et al. (2012) [26], then describe an effective statistical method for the detection of bipolar methylated genomic loci.

### Modeling bipolar methylation

We consider a genomic segment with *n* CpG sites covered fully by *m* sequence reads. Cytosines on each sequence read are labeled as either methylated or unmethylated. Therefore, the methylation data on this segment can be written as a matrix ***X***=(***X***_1_,⋯,***X***_*m*_)^*T*^, where ***X***_*i*_=(*X*_*i*1_,⋯,*X*_*in*_)^*T*^ is a vector of binary values denoting the methylation states (1 methylated, 0 otherwise) of read *i*, *i*=1,⋯,*m*. We further assume that the sequence reads are derived from *k* genomic origins, and reads from origin *l* share a methylation probability vector ***p***_*l*_,*l*=1,⋯,*k*. Similar to the mixture models [25,26] proposed for modeling ASM, we write the likelihood of observing such methylation data as

(1) $$\begin{array}{@{}rcl@{}} \prod_{i=1}^{m} L(\boldsymbol{X}_{i}|\boldsymbol{\theta}_{i})=\prod_{i=1}^{m}\left[\prod_{j=1}^{n} \theta_{ij}^{X_{ij}}(1-\theta_{ij})^{1-X_{ij}}\right], \end{array} $$

and represent the methylation probability vector as ***θ***_*i*_=***Pγ***_***i***_ where ***P***=(***p***_1_,⋯,***p***_*k*_) is a *n*×*k* matrix, and ***γ***_*i*_ is a binary vector of length *k* indicating the origin of read *i*. For example, if ***X***_*i*_ comes from origin *j*, then the *j*th entry of ***γ***_*i*_ is labeled by “1” and elsewhere is labeled by “0”. We further assume

(2) $$\begin{array}{@{}rcl@{}} \boldsymbol{\gamma}_{i}\sim\textrm{multinomial }(1,\boldsymbol{q}),\quad i=1,\cdots,m \end{array} $$

where the vector ***q*** determines the frequencies that each read comes from the *k* origins (or the proportions of the *k* origins). Based on this model, whether the methylation data show a particular pattern (homogeneous, heterogeneous or bipolar) depends not only on the origin of the reads (parameter ***γ***_*i*_,*i*=1,⋯,*m*), but also on the differentiation of the methylation probability vectors (parameter ***p***_*l*_,*l*=1,⋯,*k*) among different origins.

As a special case, for bipolar methylation, the sequence reads are assumed to come from two genomic origins (hyper-methylation or hypo-methylation) with distinct methylation probabilities. In general, the underlying epigenetic mechanisms may exhibit more complicated methylation patterns along the genome. However, due to the constraints of short read sequencing on read length and sequencing depth, we may simply concentrate on bipolar methylation for each segment and interpret more complicated epigenetic phenomena by the combination of bipolar segments. For this reason, we limit the scope of this paper to bipolar methylation (i.e. *k*=2) for each segment, although the model itself can be straightforwardly extended to the scenario of *k*>2. Another critical parameter of the model is the choice of segment length *n*. Clearly, including more CpG sites in each segment captures more salient methylation patterns, yet under a given depth of coverage, the available data contain smaller number of sequencing reads, which introduces more difficulty on discrimination. In practice, we consider *n*=4, that is, treat every four CpG sites as a segment. Nevertheless, the segment length may be adjusted to accommodate special needs in real data.

Equations (1) and (2) model the methylation data with different genomic origins (for example, different alleles or cell types) for a given locus or segment. To ultimately detect genome-wide bipolar methylated regions, a general approach is to first decide whether each segment is bipolar methylated based on the above model, then merge the identified, consecutive bipolar segments to bipolar methylated regions.

### Detecting bipolar methylated segments

From a statistical perspective, the determination of a bipolar methylated segment can be viewed as a two-sample hypothesis testing problem with group assignment unknown. Therefore, the group assignment (origin) of each read needs to be inferred first. Some literature on detecting ASM use the expectation-maximization (EM) algorithm for reads assignment and then implement model selection [26] or supervised learning from synthetic methylome [25] to identify differentially methylated segments between alleles. We note that in general, the proportion of heterogeneous epigenomes from different origins is also a unknown parameter (unlike in the ASM model where the alleles are present in equal proportions), which makes this model more complicated and often less identifiable. That is, assigning reads to hyper/hypo-methylated origins under different proportions may lead to identical likelihood. Moreover, the follow-up inference techniques in the literature, model selection or supervised learning, are not well suited for our hypothesis testing problem. A more effective statistical method is therefore desirable for detecting bipolar methylated segments.

Our strategy for deciding whether a segment is bipolar methylated is through a two-step approach: first clustering the reads into two groups, then testing whether the two groups share the same mean. In particular, suppose the *m* reads are clustered into a hyper-methylation group $\mathcal {G}_{1}$ and a hypo-methylation group $\mathcal {G}_{2}$, with mean ***p*** ^(1)^ and ***p*** ^(2)^, respectively. We would like to test *H*_0_:***p*** ^(1)^=***p*** ^(2)^ versus *H*_*a*_:***p*** ^(1)^>***p*** ^(2)^. To further take into account the bipolar behavior, we may modify the null and alternative hypotheses as: $H_{0}: 0\le p_{i}^{(1)}-p_{i}^{(2)}\le \tau, \forall i$ versus $H_{a}: p_{i}^{(1)}-p_{i}^{(2)}>\tau, \forall i$. The parameter *τ* plays a role in controlling the separation of bipolar groups: the larger it is, the more conservative the test is on determining whether the segment is bipolar methylated. The detailed detection procedure is described as following.

Step 1: Allocate the sequence reads into hyper/hypo-methylated groups using nonparametric Bayesian clustering.

- Allocate *m* reads to different *clusters* using the DPM search method [24]. This method adopts a fast search algorithm to find the maximum a posteriori (MAP) solution (the most likely cluster assignments) to a DPM model for the methylation data. We provide details for the DPM model in Additional file 1 of Supplementary Material.
- We define a bipolar threshold parameter *δ* for the methylation probabilities on the CpG sites. For clusters satisfying the bipolar criterion below, allocate their reads to two *candidate groups*. Mathematically, suppose *k* clusters $\mathcal {C}_{1},\cdots,\mathcal {C}_{k}$ are generated in the previous step, and $\mathcal {C}_{i}=\{\boldsymbol {r}_{i1},\cdots,\boldsymbol {r}_{{im}_{i}}\},1\le i\le k$ where ***r***_*ij*_ is the *j*th read in the *i*th cluster. Denote the mean of cluster *i* by $\boldsymbol {c}_{i}=\frac {1}{m_{i}}\sum _{j=1}^{m_{i}}\boldsymbol {r}_{\textit {ij}}=(c_{i1},\cdots,c_{\textit {in}})^{T}$. Then candidate group 1 is defined as $\mathcal {H}_{1}=\{\boldsymbol {r}_{\textit {ij}}:c_{\textit {il}}\le \delta,1\le l\le n\}$ where *δ* is a pre-specified parameter. Similarly, the other candidate group is defined as $\mathcal {H}_{2}=\{\boldsymbol {r}_{\textit {ij}}:c_{\textit {il}}\ge 1-\delta,1\le l\le n\}$. Clearly, $\mathcal {H}_{1}$ and $\mathcal {H}_{2}$ are separated by at least 1−2*δ* at each CpG site.
- For clusters which do not satisfy the bipolar criterion, allocate their reads to the candidate groups based on their distances (e.g., Euclidean) to the candidate group means (i.e., equivalent to using the maximum likelihood discriminant rule). The procedure in Steps 1(b) and 1(c) reduces the clusters into two *bipolar groups*. Using mathematical notation, suppose the means of $\mathcal {H}_{1}$ and $\mathcal {H}_{2}$ are denoted by ***h***_1_ and ***h***_2_, respectively, then bipolar group 1 is defined as $\mathcal {G}_{1}=\mathcal {H}_{1}\cup \{\boldsymbol {r}_{\textit {ij}}:\boldsymbol {r}_{\textit {ij}}\notin \mathcal {H}_{1}\cup \mathcal {H}_{2}, d(\boldsymbol {r}_{\textit {ij}},\boldsymbol {h}_{1})<d(\boldsymbol {r}_{\textit {ij}},\boldsymbol {h}_{2})\}$, and similarly, bipolar group 2 is defined as $\mathcal {G}_{2}=\mathcal {H}_{2}\cup \{\boldsymbol {r}_{\textit {ij}}:\boldsymbol {r}_{\textit {ij}}\notin \mathcal {H}_{1}\cup \mathcal {H}_{2}, d(\boldsymbol {r}_{\textit {ij}},\boldsymbol {h}_{1})>d(\boldsymbol {r}_{\textit {ij}},\boldsymbol {h}_{2})\}$.

Step 2: Calibrate the separation of hyper/hypo-methylated groups by hypothesis testing. Depending on the depth of coverage, nonparametric (e.g., permutation) or parametric (e.g., Wald or likelihood ratio) test may be employed. For example, if using permutation test, we need to first define an inter/intra-group-distance test statistic (e.g., inverse of the Davies-Bouldin index), and randomly permute group identity for the reads to obtain its null distribution. Then we calculate p-value using the observed statistic and the null distribution.

We add three important remarks below to further elucidate the proposed method. 1) The clustering step is essentially equivalent to the E-step in the EM algorithm [25,26] which estimates membership for each read. However, because of the less restricted model assumption on the proportion of epigenomic origins, we do not intend to maximize the likelihood iteratively but rather adopt a direct, heuristic clustering for reads assignment. The clustering, as well as the whole bipolar detection performance will be illustrated by simulation study (see Simulation III: Comparing DPM with *k*-means and Bayesian mixture clustering). 2) We note that the parameter *δ* should not be confused with *τ*. Although they both help control the separation of bipolar groups, *δ* acts as a threshold for choosing candidate groups whereas the boundary between final bipolar groups can be blurred by reads not belonging to candidate groups. In practice, when the number of reads *m* is small, it may be difficult to set appropriate *δ* value to find candidate groups in Step 1(b). As an alternative, we can adopt *k*-means (*k*=2) clustering to the cluster means obtained in step 1(a) to form the bipolar groups. 3) In the testing step, due to limited sequencing depth, most bisulfite sequencing data do not satisfy the conventional “large sample” assumption for parametric testing. For this reason, permutation test may be more preferable in the second step. The test statistic is chosen to best characterize the separation of bipolar groups.

### Simulation study

We perform three simulation studies. Simulation I is to assess the type-I error and power of the proposed method under different settings of parameters. First, we generate methylation states on a segment of 4-CpG sites using the hierarchical model introduced in Methods. Guided by prior knowledge obtained from a recent comprehensive genome-wide study with methylomes derived from 17 mouse tissues [28] (see Additional file 2 in Supplementary Material for details), we set the following methylation probabilities in the simulation study. Under the null hypothesis (non-bipolar, i.e., one cell-type), all the methylation probabilities are sampled from beta(8, 8), with mean 0.5 and standard deviation 0.12. Under the alternative hypothesis (bipolar, i.e., two cell-types), the methylation probabilities of the hyper-methylated group and the hypo-methylated group are sampled from beta(5.96, 0.89) and beta(0.72, 4.42), respectively. The methylation probabilities of these two groups vary around means 0.87 (hyper-methylated) and 0.14 (hypo-methylated) with standard deviations 0.12 and 0.14, respectively. We then apply the proposed method to test whether the segment is bipolar methylated or not, for a pre-specified *δ* and *τ*. Repeating this procedure, we calculate the type-I error rate (false positive rate) and power (true positive rate). To get a complete evaluation of the detection method under different settings, these simulations are performed using different number of reads (small, moderate and large) and different cell-type proportions (from low to high). In Simulation II, we further examine the p-values from bipolar testing on a 4-CpG segment with 16 reads. This simulation study provides us an impression about how the bipolar decision varies with the number of reads taking on methylation patterns (0,0,0,0) and (1,1,1,1), for different thresholds *δ*. In Simulation III: Comparing DPM with *k*-means and Bayesian mixture clustering, we compare the DPM clustering method with two other clustering methods: *k*-means and Bayesian mixture clustering, in terms of mis-classification rate of reads assignment and power of bipolar detection under the alternative hypothesis. The purpose is to illustrate that the DPM method, as a likelihood-based clustering method, is more suitable than conventional distance-based clustering methods in handling methylation data (i.e., binary vector) clustering.

### Real data sets

To further evaluate the performance of our method on real data, we mix sorted cells to generate heterogeneous cell populations for two previously published data sets (the mouse brain data and the human blood data) [19,29] separately and re-analyze them. The human blood data sets consist of methylomes from human B cells and neutrophils, and the mouse brain data sets consist of methylomes from mouse neuron and glia cells. Two artificial datasets, human blood mixed dataset and mouse brain mixed dataset, are created by pooling all reads for human B cells and neutrophils together and pooling all reads for mouse neuron and glia cells together, respectively. We then perform sequence read processing as previously described [29]. In short, low quality bases are trimmed and adapter sequences are removed. The processed reads are aligned to the corresponding reference genomes (mm10 or hg19) using Bismark [6] with parameters “-n 2 -l 50”. For each data set, the methylation patterns of segments containing four neighboring CpG dinucleotides are extracted. Our choice of four-CpG segments for pattern analysis is based on the practical concern on the read length and coverage of the current methylome data. As mentioned before, using a length threshold with less CpG sites will result in more segments but reduce the capacity to capture the complexity of the pattern variations. On the other hand, the more CpG sites are encompassed in a segment, the higher complexity of pattern variations will be shown in the segment, but at the same time, the fewer segments in the genome can be analyzed. For comparison purpose, we first identify two sets of DMRs for neuron-glia cell pair and neutrophil-B cell pair, using the segments with at least 10Xs read coverage from original sorted cells. We then generate two artificially pooled data sets for blood and brain, and predict CSM regions from the pooled data sets using the proposed method. The predicted CSMs using pooled samples are compared with DMRs identified using paired samples.

## Results

### Simulation study

#### Simulation I: Type-I error and power of the proposed method

As described in Methods, we generated methylation states on a segment of 4-CpG sites under the null and alternative hypotheses, for different number of reads and different cell-type proportions. The number of reads are set to be *m*=10, 20, and 100, corresponding to low, moderate, and high sequencing depth, respectively. The cell-type proportions *w*=10*%*,20*%*,30*%*,40*%*,50*%*, varying from unbalanced to balanced. Applying the proposed bipolar detection method with pre-specified *δ* and *τ*, we calculated p-values for the simulated data and obtained empirical type-I error rate and power based on 5,000 simulations. Table 1 lists the results when setting *δ*=0.35, *τ*=0 and 0.32. From the table, it can be seen that in general, for each cell-type proportion, the proposed method achieves more power as the number of reads increases. On the other hand, for each number of reads, the bipolar pattern becomes more distinguishable as the cell-type proportion changes from unbalanced (10%) to balanced (50%). Comparing the results for *τ*=0 and *τ*=0.32, we see that, when *τ* is set to a larger value (i.e., test whether the bipolar groups are separated by a higher threshold), the method may lose power slightly but the type-I error can be better controlled. In other words, the method becomes more conservative for larger *τ*. More sensitivity analysis on setting *δ* and *τ* for this simulation study can be found in Additional file 3 of Supplementary Material. In real data analysis, parameter *τ* can be chosen using prior knowledge obtained from DMR analysis (see for example Additional file 2 in Supplementary Material).

**Table 1:** **Empirical type-I error rate and power for bipolar methylation detection**

***τ*** **=0**	**Type-I error** ^**∗**^			**Power**		
	***m*** **=10**	***m*** **=20**	***m*** **=100**	***m*** **=10**	***m*** **=20**	***m*** **=100**
*w*=10*%*	.079	.075	.087	.279	.580	.983
*w*=20*%*	.090	.077	.082	.590	.875	.997
*w*=30*%*	.082	.080	.094	.875	.976	.998
*w*=40*%*	.084	.083	.088	.887	.986	.997
*w*=50*%*	.085	.088	.088	.931	.995	.998
***τ*** **=0** ***.*** **32**	**Type-I error** ^**∗**^			**Power**		
	***m*** **=10**	***m*** **=20**	***m*** **=100**	***m*** **=10**	***m*** **=20**	***m*** **=100**
*w*=10*%*	.032	.015	.006	.275	.556	.871
*w*=20*%*	.034	.015	.004	.528	.771	.984
*w*=30*%*	.031	.017	.008	.770	.937	.996
*w*=40*%*	.027	.020	.008	.778	.946	.996
*w*=50*%*	.025	.016	.006	.782	.951	.997

The empirical type-I error rate and power are calculated from 5,000 simulations under significance level 0.05, for different number of reads *m* and for different cell-type proportion *w*. In all simulations, we set the threshold parameter *δ*= 0.35. The type-I error rates^**∗**^ for the same *m* but different *w* are not the same because we set different methylation probability vectors for different *w* when generating data under *H*_0_, although these probabilities are all sampled from beta(8, 8).

#### Simulation II: Testing of bipolar methylation on various patterns

In order to better illustrate how the threshold *δ* controls the decision of bipolar methylation, we conducted another simulation study. In this simulation, we considered all possible methylation patterns on a 4-CpG segment with 16 reads. Denote the number of reads with methylation pattern (0,0,0,0) by *i*, and the number of reads with methylation pattern (1,1,1,1) by *j*. We randomly generated (16−*i*−*j*) reads with other methylation patterns (there are 14 patterns left). For methylation patterns generated under different (*i*,*j*) settings, we applied the proposed method using *τ*=0.32 and different *δ* values to decide whether the segment is bipolar methylated or not and reported the corresponding p-values. For each (*i*,*j*) setting, we repeated the simulation 100 times, and listed in Table 2 the average p-values based on the 100 repetitions. We see a clear trend from the table that as the bipolar patterns vary from weak (*i*=1,*j*=1) to strong (*i*=8,*j*=8) in a 4-CpG segment with 16 reads, the p-values decreases gradually, which indicates an increasing amount of evidence on rejecting the null (non-bipolar) hypothesis. On the other hand, the bipolar detection results are quite stable over different *δ* settings. In particular, the “boundary” patterns (*i*,*j*) for calling this 4-CpG segment with 16 reads as bipolar methylated appear to be: (1,12),(2,8) and (3,5).

**Table 2:** **Average p-values for bipolar methylation detection**

**(** ***i*** **,** ***j*** **)**	***δ***					**(** ***i*** **,** ***j*** **)**		***δ***				
	**0.2**	**0.25**	**0.3**	**0.35**	**0.4**			**0.2**	**0.25**	**0.3**	**0.35**	**0.4**
(1, 1)	1	1	1	1	1		(3, 7)	**0.03**	**0.02**	**0.02**	**0.01**	**0.02**
(1, 2)	1	1	1	1	1		(3, 8)	**0.02**	**0.01**	**0.01**	**0.01**	**0.02**
(1, 3)	0.95	0.90	0.95	0.99	0.97		(3, 9)	**0.01**	**0.01**	**0.01**	**0.01**	**0.01**
(1, 4)	0.82	0.71	0.76	0.76	0.81		(3,10)	**0**	**0**	**0**	**0.01**	**0**
(1, 5)	0.58	0.69	0.73	0.70	0.64		(3,11)	**0**	**0**	**0**	**0**	**0**
(1, 6)	0.51	0.47	0.58	0.50	0.57		(3,12)	**0**	**0**	**0**	**0**	**0**
(1, 7)	0.46	0.34	0.29	0.42	0.36		(3,13)	**0**	**0**	**0**	**0**	**0**
(1, 8)	0.23	0.29	0.25	0.27	0.35		(4, 4)	**0.05**	**0.04**	**0.03**	**0.03**	**0.04**
(1, 9)	0.14	0.14	0.15	0.15	0.16		(4, 5)	**0.02**	**0.02**	**0.01**	**0.02**	**0.01**
(1,10)	0.09	0.09	0.11	0.08	0.09		(4, 6)	**0.01**	**0.01**	**0.01**	**0.01**	**0.01**
(1,11)	0.07	0.07	0.06	0.06	0.07		(4, 7)	**0**	**0**	**0**	**0**	**0**
(1,12)	**0.05**	**0.04**	**0.05**	**0.05**	**0.05**		(4, 8)	**0**	**0**	**0**	**0**	**0**
(1,13)	**0.02**	**0.02**	**0.03**	**0.03**	**0.04**		(4, 9)	**0**	**0**	**0**	**0**	**0**
(1,14)	**0**	**0**	**0**	**0**	**0**		(4,10)	**0**	**0**	0	**0**	**0**
(1,15)	**0**	**0**	**0**	**0**	**0**		(4,11)	**0**	**0**	**0**	**0**	**0**
(2, 2)	0.72	0.79	0.81	0.60	0.61		(4,12)	**0**	**0**	**0**	**0**	**0**
(2, 3)	0.56	0.59	0.48	0.41	0.44		(5, 5)	**0**	**0**	**0**	**0**	**0**
(2, 4)	0.38	0.41	0.45	0.28	0.28		(5, 6)	**0**	**0**	**0**	**0**	**0**
(2, 5)	0.23	0.20	0.24	0.16	0.15		(5, 7)	**0**	**0**	**0**	**0**	**0**
(2, 6)	0.14	0.13	0.10	0.11	0.07		(5, 8)	**0**	**0**	**0**	**0**	**0**
(2, 7)	0.06	0.08	0.07	**0.05**	0.06		(5, 9)	**0**	**0**	**0**	**0**	**0**
(2, 8)	**0.05**	**0.05**	**0.05**	**0.04**	**0.04**		(5,10)	**0**	**0**	**0**	**0**	**0**
(2, 9)	**0.03**	**0.03**	**0.03**	**0.04**	**0.03**		(5,11)	**0**	**0**	**0**	**0**	**0**
(2,10)	**0.03**	**0.03**	**0.03**	**0.02**	**0.02**		(6, 6)	**0**	**0**	**0**	**0**	**0**
(2,11)	**0.02**	**0.01**	**0.02**	**0.01**	**0.02**		(6, 7)	**0**	**0**	**0**	**0**	**0**
(2,12)	**0**	**0.01**	**0.01**	**0.01**	**0.01**		(6, 8)	**0**	**0**	**0**	**0**	**0**
(2,13)	**0**	**0**	**0**	**0**	**0**		(6, 9)	**0**	**0**	**0**	**0**	**0**
(2,14)	**0**	**0**	**0**	**0**	**0**		(6,10)	**0**	**0**	**0**	**0**	**0**
(3, 3)	0.23	0.31	0.11	0.16	0.15		(7, 7)	**0**	**0**	**0**	**0**	**0**
(3, 4)	0.16	0.12	0.08	0.08	0.10		(7, 8)	**0**	**0**	**0**	**0**	**0**
(3, 5)	0.06	**0.04**	**0.05**	**0.05**	**0.04**		(7, 9)	**0**	**0**	**0**	**0**	**0**
(3, 6)	**0.04**	**0.03**	**0.03**	**0.03**	**0.04**		(8, 8)	**0**	**0**	**0**	**0**	**0**

The average p-values are calculated from 100 simulations for a 4-CpG segment with 16 reads, using *τ*= 0.32 and different *δ* values. Significant p-values (under significance level 0.05) are marked with boldfaced font. Zero-valued p-values are actually <5 × 10^-3^. In each simulation, we generate *i* reads with methylation pattern (0,0,0,0), *j* reads with methylation pattern (1,1,1,1), and randomly generate (16-*i*-*j*) reads with other methylation patterns.

#### Simulation III: Comparing DPM with *k*-means and Bayesian mixture clustering

**Figure 2:**
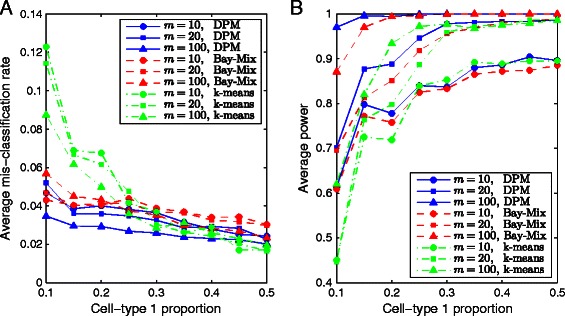
**Comparison between DPM search,** ***k*** **-means and Bayesian mixture clustering in Simulation III.** **A**. Average mis-classification rates. **B**. Average power. These results are obtained from 1,000 simulations under the alternative hypothesis, for different number of reads and for different cell-type proportions, using DPM search, *k*-means and Bayesian mixture clustering.

We performed a comparison study between DPM, *k*-means and Bayesian mixture clustering to illustrate the advantage of likelihood-based method as compared to distance-based method for clustering data of binary vectors. In this simulation study, methylation data were generated under the alternative hypothesis for different number of reads and different cell-type proportions, using the same settings as in Simulation I. We applied three clustering methods to the simulated data: DPM search, *k*-means and finite mixture clustering via Bayesian inference, and calculated their corresponding mis-classification rates and power. The average mis-classification rates based on 1,000 simulations are shown in Figure 2A. Clearly, as the cell-type proportion *w* changes from unbalanced (10%) to balanced (50%), all three methods show decreasing average mis-classification rates. As the number of reads *m* increases, the average mis-classification rate decreases. Comparing the three clustering methods, we see that for almost all settings of *m* and *w*, DPM is able to achieve more accurate clustering results than the other two. The empirical power results for detecting bipolar loci using “DPM+hypothesis testing”, “*k*-means+hypothesis testing” and “Bayesian mixture clustering+hypothesis testing” are shown in Figure 2B, from which we see that larger number of reads or more balanced cell-type proportion results in higher power. Again, “DPM+hypothesis testing” outperforms the other two for almost all settings of *m* and *w*.

### Application on public data sets

#### Identified CSMs in mouse neuron and glia cells

The methylomes for the frontal cortex of 12-month mice [29] were downloaded from NCBI Gene Expression Ominibus (GSE47966). The bisulfite sequencing data sets were generated from populations of nuclei obtained by fluorescence-activated cell sorting to enrich neurons (NeuN+) or glia (NeuN-). We started with 636.3 M reads for neuron and 420.5 M reads for glia. There were 182,046 and 93,715 4-CG segments covered with at least 10 reads identified for neuron and glia, respectively. After filtering the segments with a recently reported mouse allelic-specific methylation map [17], we obtained a common set of 42,087 segments with at least 10Xs read coverage in both neuron and glia. Interestingly, we found that 84.0% of the imprinted CpGs (605 out of 720 with at least 10Xs coverage) exist in bipolar methylated segments.

**Figure 3:**
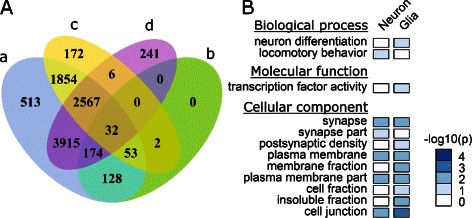
**Analysis of mouse brain methylomes.** **A**. Venn diagram shows the relationships between (a) bipolar segments identified from pooled dataset, (b) DMR identified between neuron and glia, (c) bipolar segments identified from glia, and (d) bipolar segments identified from neuron. **B**. Gene ontology analysis of genes associated with bipolar methylated segments in neuron and glia datasets, respectively. P-values for GO enrichment were adjusted with Bonferroni correction.

We next focused on the common segments in the following analysis. Using the proposed method, 6,935 and 4,686 segments were identified as bipolar methylated in neuron and glia data sets, respectively. Meanwhile, 9,236 segments from the pooled data set were identified to be bipolar methylated. On the other hand, differentially methylated CpGs (DM-CpGs) between neuron and glia cells were identified by using Fisher’s Exact Test (5% FDR). A four-CG segment was considered as differentially methylated if all the four CpG sites are significantly differentially methylated in the same direction. Based on this criterion, a total of 389 segments from the common segments were identified as differentially methylated between neuron and glia. Out of these differentially methylated segments, 387 (99.5%) were predicted as bipolar methylated from the pooled data set, indicating that our model can recover nearly all of the real CSMs between the major cell types in brain (Figure 3A). Not surprisingly, most of the bipolar methylated segments in glia (96.2%) and neuron (96.4%) were also predicted as bipolar methylated in pooled data set.

The identification of bipolar methylated regions provides a novel and efficient approach to annotate genomic regions under differential methylation regulation within a cell population. To examine the functional relevance, we performed GO analysis using DAVID bioinformatics resources [30] for genes containing bipolar methylated segments. Gene structure annotations were retrieved from UCSC genome browser [31], and gene region was defined to be from transcription start site to transcription end site. The aforementioned common segments from the above analysis were mapped to 5,858 genes, among which 1,548 and 1,008 genes contain bipolar methylated segments in neuron and glia cells, respectively. GO enrichment analysis shows that neuron and glia share some cell-specific methylated genes, which in particular, may control activities executed at plasma membrane fraction and cell junction (Figure 3B). Interestingly, genes involved in locomotory behavior and synapse functions are enriched in the gene list of the predicted CSMs within neuron subpopulation. It has been reported that locomotory nervous system consists of several classes of interneurons and motor neurons, and these neurons communicate with each other via synapses [32,33]. Further studies would be highly desirable to validate whether the CSM loci we predicted are differentially methylated between these neuron subsets.

#### Identified CSMs in human neutrophil and B cells

**Figure 4:**
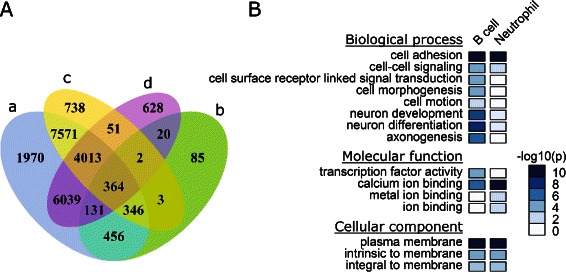
**Analysis of human blood methylomes.** **A**. Venn diagram shows the relationships between (a) bipolar segments identified from pooled dataset, (b) DMR identified between B cell and neutrophil, (c) bipolar segments identified from B cell, and (d) bipolar segments identified from neutrophil. **B**. Gene ontology analysis of genes associated with bipolar methylated segments in B cell and neutrophil datasets, respectively. P-values for GO enrichment were adjusted with Bonferroni correction.

The methylomes for neutrophil and B cells of human [19] were downloaded from NCBI Gene Expression Ominibus (GSE31971). For this data set, 712,214 and 654,506 4-CG segments covered by at least 10 reads were identified in B cell and neutrophils, respectively. Segments overlapped with imprinted CpGs predicted in previous study [26] were discarded, and finally we got 183,966 segments which are covered by at least 10 reads in both data sets. Out of these 183,966 common segments, 13,088 and 11,248 segments were identified as bipolar methylated in B cell and neutrophil data sets, respectively. Meanwhile, 20,890 segments were identified to be bipolar methylated from the pooled data set. A total of 1,407 differentially methylated segments between B cell and neutrophils were identified using the same procedure as described in the previous section. Among these 1,407 differentially methylated segments, 1,297 (92.2%) were covered in the predicted bipolar methylated segments from pooled human blood data set (Figure 4A). Similarly, most of the bipolar segments identified from B cell (93.9%, 12,294 from 13,088) and from neutrophils (93.8%, 10,547 from 11,248) were included in the sets of bipolar methylated segments identified from pooled data.

The aforementioned common segments were mapped to 13,065 genes, among which, the promoter regions of 2,607 and 2,017 genes are associated with bipolar methylated segments in B cells and neutrophils, respectively. GO functional analysis indicated that these genes are enriched for functions including cell adhesion, cell-cell signaling, etc (Figure 4B). For multicellular organisms, cell adhesion is critical for tissue formation during morphogenesis. For both neutrophils and B cells, we found that the most significantly enriched GO category of “molecular function” is calcium ion binding. The regulation of cytosolic concentration of calcium ions (Ca^2+^) is of vital importance for the development and function of B cells [34] and for the activation of neutrophils [35,36]. It has been suggested that many neuron development genes are probably silenced by DNA methylation in B cells [37]. Some of these neuron development genes are important for blood development as well. For examples, B cells express receptors for brain-derived neurotrophic factor (BDNF), which is critical for normal B lymphocyte development through paracrine effects in the bone marrow [38]. Neuropilin 2 (NRP2), a receptor for the vascular endothelial growth factor (VEGF), is also found to be important for the growth of midbrain dopaminergic axons [39]. In summary, the real data analyses indicate that the method we developed can not only reveal the differentially methylated regions identified with traditional approach but also uncover genomic regions under epigenetic regulation in subpopulations.

## Discussion and conclusions

We developed a statistical approach to detect bipolar DNA methylation in bisulfite sequencing data, through nonparametric Bayesian clustering and hypothesis testing. Simulation studies demonstrated that our method achieves high specificity under all settings. The sensitivity increases as cell-type mixture proportion becomes more balanced and as depth of coverage increases. With allele-specific events filtered out, this approach can be used to identify cell-specific genes/pathways under epigenetic control within a heterogeneous cell population. We applied the method to analyze data from mouse brain and human blood methylomes. Results on real data analysis demonstrated that the predicted CSMs are highly consistent with the DMRs identified by using purified neuron-glia cells and neutrophil-B cells.

Methylation differences observed among sequence reads mapped to the same locus may result from: 1) allele specific DNA methylation; 2) asymmetric DNA methylation; and 3) cell-subset specific DNA methylation. ASM may be classified into imprinted (parent-of-origin dependent ASM) and sequence dependent ASM. Recent genome analyses revealed 55 discrete genomic loci imprinted in the mouse genome [17] and 51 loci imprinted in the human genome [40]. Meanwhile, the majority of sequence dependent ASM CpG sites are scattered throughout the genome [17]. The asymmetric DNA methylation can be determined with the genome-wide hairpin bisulfite sequencing technique recently developed by our lab [41]. We found that 88.5% and 91.9% CpG sites sequenced for mouse renewing and differentiating ES cells are symmetrically methylated, respectively. In addition, the asymmetric methylated CpG sites are frequently associated with random DNA methylation patterns resulting from stochastic DNA methylation events [12]. Thus, after the filtering of imprinted loci, bipolar methylated regions identified in this study are likely to be cell-subset specifically methylated.

Our approach provides new insights to the identification and interpretation of partially methylated genomic loci. It is advantageous over currently available methods for CSM identification in two aspects. 1) The detection of bipolar DNA methylation is based on bisulfite sequencing data from a heterogeneous cell population, while traditional CSM identification relies on detecting DMRs, i.e., testing for differential methylation levels between highly purified cell subsets. The proposed method thus provides a feasible solution to the analysis of DNA methylation in higher organisms where cells are difficult to be dissociated. Apropos, a growing body of evidence from single cell analysis reveals significant cellular heterogeneity even within a cloned population. Therefore, CSM regions identified by our method generally include not only DMRs but also partially methylated genomic regions caused by the same type of cells at different stages, and the latter are of particular interest as they cannot be detected by traditional methods. 2) Besides identifying CSM regions, the proposed method simultaneously estimates the proportions of heterogeneous epigenomes in bisulfite sequencing data. In recently published literature, some efforts have been made to estimate the proportions of different cell types in unfractionated samples by employing the identified DMRs as markers of cell identity. For example, Guintivano et al. (2013) [42] incorporated cell epigenotype specific (CETS) markers in a linear slope model to quantify neuronal and glial proportions. Houseman et al. (2012) [43] and Montaño et al. (2013) [44] employed DMRs and adopted a regression calibration model to estimate the proportions of different cell types in unfractionated blood sample and in brain tissue, respectively. Compared to the prediction by identified DMRs, our method provides a straightforward solution to this problem.

Our method for detecting bipolar DNA methylation is also related to statistical methods recently developed for ASM prediction. In particular, Fang et al. (2012) [26] proposed a model selection method to identify ASM by comparing Bayesian information criterion (BIC) under non-allele specific and allele-specific models. Peng and Ecker (2012) [25] adopted supervised learning to classify candidate regions into ASM or non-ASM by using features (estimated methylation levels and allele frequencies) extracted from a mixed model. Compared with these two methods, our bipolar detection framework has several advantages: 1) Our model makes no restriction on the ratio between number of hyper-methylated and hypo-methylated reads, therefore, it is more appropriate for predicting CSM. 2) By treating bipolar detection as a hypothesis testing problem, our method is more suitable for sensitivity and specificity evaluation than the BIC-based model selection method [26] and the supervised learning method [25]. 3) Because neither EM algorithm nor Markov chain Monte Carlo (MCMC) is required, the DPM search algorithm is easy to implement and requires the least amount of computation load.

The method developed in this study will greatly extend our capacity to dissect the epigenetic heterogeneity in a cell population, in particular for the ones with limited or no prior knowledge of cell-type composition. Detecting bipolar methylation patterns is an important step for unlocking the biological meaning of epigenetic heterogeneity; however, the methodology development is still in initial stages. Due to the limitation on depth of coverage and read length, with current bisulfite sequencing data, bipolar DNA methylation can only be detected in small segments (e.g., 4-CpG). In general, more complicated methylation patterns arising from a mixture of various cell types or cells at various stages may be decomposed into a combination of bipolar DNA methylation patterns presented at multiple loci. Our future work will be directed to investigate the functional relevance of partially methylated genomic loci and incorporate their combinations as epigenetic signatures for complex phenotypic and functional variability.

## Availability of supporting data

The methylomes of 17 adult mouse tissues are publicly available from NCBI Gene Ominibus (GSE42836). The mouse brain methylomes of neuron and glia cells are publicly available from NCBI Gene Expression Ominibus (GSE47966). The human blood methylomes of neutrophil and B cells are publicly available from NCBI Gene Expression Ominibus (GSE31971).

## Additional files

Additional file 1
**DPM search method.**

Additional file 2
**Summary and descriptive statistics for hyper/hypo-methylated groups in published data sets [** 28 **].**

Additional file 3
**More results from Simulation I on type-I error and power.**
