# Supplementary material for: Nonparametric Bayesian clustering to detect bipolar methylated genomic loci
Source: BMC Bioinformatics. 2015 Jan 16;16:11. doi: 10.1186/s12859-014-0439-2 (PMC4302125; doi:10.1186/s12859-014-0439-2)
Supplement: Additional file 1 — DPM search method. [file 12859_2014_439_MOESM1_ESM.pdf]

Supplementary Materials

Additional file 1: DPM search method

We adopt a nonparametric Bayesian Dirichlet process mixture (DPM) model to clustering the m sequence reads. Denote by $\{\boldsymbol{X}_i, i = 1, \cdots, m\}$ the m independent sequence reads, with $\boldsymbol{X}_i = (X_{i1}, X_{i2}, \cdots, X_{in})^T$ a binary vector at n sites. The DPM model can be written as:

$$\begin{aligned} X_{ij} &\sim \text{Bernoulli}(p_{ij}),\ j = 1, \ldots, n,\ i = 1, \ldots, m \\ \boldsymbol{p}_i &= (p_{i1}, \ldots, p_{in})^T \\ \boldsymbol{p}_i &\sim G, \quad G \sim \text{DP}(\alpha, G_0), \\ G_0 &= \prod_{j=1}^{n} G_{0j},\ G_{0j} \sim \text{Beta}(\alpha_j, \beta_j), \end{aligned}$$

where G is a random distribution of $\boldsymbol{p}_i$ which is given a Dirichlet process (DP) prior with concentration parameter α and base measure G_0. The base measure G_0 is formed by independent Beta distributions with pre-specified prior parameters $\{(\alpha_j, \beta_j), j = 1, \cdots, n\}$. The DP prior has the effect of grouping similar $\boldsymbol{p}_i$'s into clusters hence is widely used for clustering.

While the posterior inference of the above model can be achieved through Markov chain Monte Carlo sampling, these sampling methods are often slow to implement. In this work, we employ a fast search algorithm to find the maximum a posteriori (MAP) solution, following the method of Daumé III (2007)[24]. The main idea is to set a score function, and sequentially assign the ordered data points $\{\boldsymbol{X}_i, i = 1, \cdots, m\}$ either to one of the existing clusters or to a new cluster based on the score function. We choose a fast in-admissible score function which has demonstrated extremely good performance on MAP search. In our algorithm, we need to determine the marginal join likelihood: $H(\boldsymbol{X}_S) = \int f(\boldsymbol{X}_S|\boldsymbol{p})dG_0(\boldsymbol{p})$ and a conditional probability of a new $\boldsymbol{X}$ conditional on $\boldsymbol{X}_S$: $H(\boldsymbol{X}|\boldsymbol{X}_S) = H(\boldsymbol{X}, \boldsymbol{X}_S)/H(\boldsymbol{X}_S)$, for $\boldsymbol{X}_S = \{\boldsymbol{X}_i : i \in S\}$ and S is an index set. Here f is the joint likelihood of $\boldsymbol{X}_S$ assuming that the elements in $\boldsymbol{X}_S$ are independent Bernoulli with a common parameter $\boldsymbol{p}$. Due to the fact that the Beta prior is conjugate to Bernoulli likelihood, we can calculate $H(\boldsymbol{X}_S)$ and $H(\boldsymbol{X}|\boldsymbol{X}_S)$ analytically.
