# Supplementary material for: Nonparametric Bayesian clustering to detect bipolar methylated genomic loci
Source: BMC Bioinformatics. 2015 Jan 16;16:11. doi: 10.1186/s12859-014-0439-2 (PMC4302125; doi:10.1186/s12859-014-0439-2)
Supplement: Additional file 2 — Summary and descriptive statistics for hyper/hypo-methylated groups in published data sets [ 28 ]. [file 12859_2014_439_MOESM2_ESM.pdf]

Supplementary Materials

Additional file 2: Summary and descriptive statistics for hyper/hypo-methylated groups in published data sets

We used the hyper/hypo-methylated group information obtained from Hon et al. (2013)[27] to guide our parameter settings in Simulations I and III in **Methods**. These published data sets of methylomes in 17 adult mouse tissues at shallow coverage were downloaded from NCBI Gene Ominibus (GEO, accession number GSE42836). We extracted the coordinates of the identified tissue-specific differentially methylated regions (tsDMRs) and their methylation pattern for 16 samples (placenta sample is excluded because it is globally hypo-methylated). The distribution of methylation levels in hyper/hypo-methylated tsDMRs for all samples combined is shown in Supplementary Figure 1, with summary statistics listed in Supplementary Table 1. Supplementary Figure 2 depicts the distribution of methylation levels in hyper/hypo-methylated tsDMRs for each sample. The distribution of the differences in methylation for the identified tsDMRs is shown in Supplementary Figure 3, and the absolute methylation differences between neuron and glia for differentially methylated CpGs (DM-CpGs) are plotted in Supplementary Figure 4.

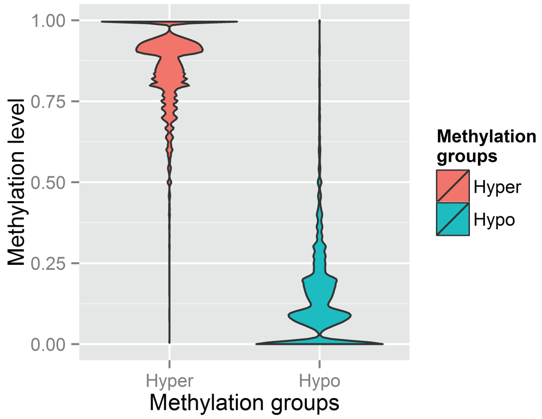

Supplementary Figure 1: Distribution of methylation levels in hyper/hypo-methylated tsDMRs for combined samples.

Supplementary Table 1: Summary statistics of methylation levels in hyper/hypo-methylated tsDMRs for combined samples.

	# CpG sites	Min	1st Qu.	median	Mean	Std	3rd Qu.	Max
Hypo group	654,233	0.00	0.00	0.01	0.14	0.14	0.20	1.00
Hyper group	1,642,007	0.00	0.81	0.90	0.87	0.12	0.95	1.00

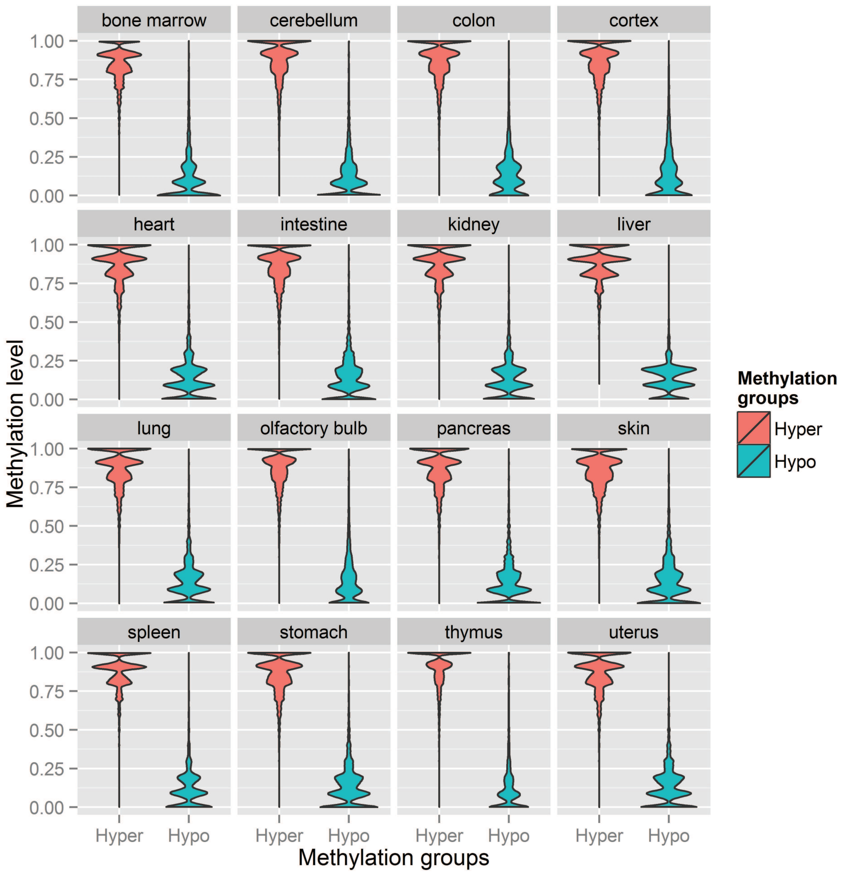

Supplementary Figure 2: Distribution of methylation levels in hyper/hypo-methylated tsDMRs for combined each sample.

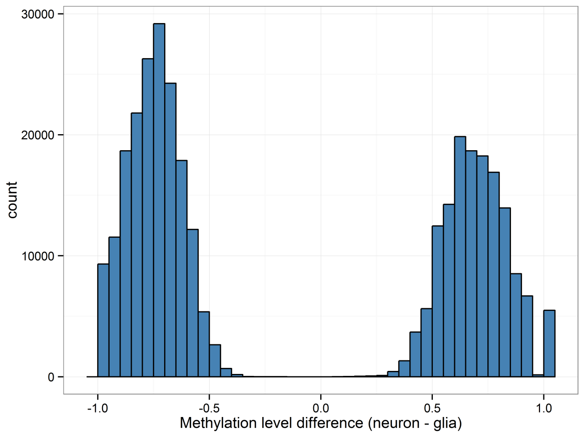

Supplementary Figure 3: Distribution of methylation level differences between neuron and glia for tsDMRs identified by using Fisher's Exact Test.

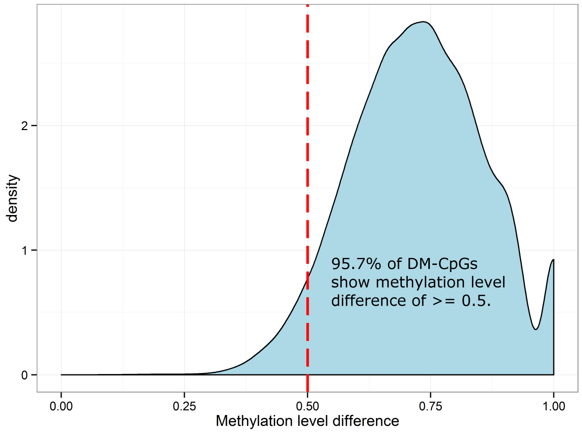

Supplementary Figure 4: Distribution of methylation level differences between neuron and glia cells shows that the majority of DM-CpGs are of methylation level difference ≥ 0.5.
