# Supplementary material for: Nonparametric Bayesian clustering to detect bipolar methylated genomic loci
Source: BMC Bioinformatics. 2015 Jan 16;16:11. doi: 10.1186/s12859-014-0439-2 (PMC4302125; doi:10.1186/s12859-014-0439-2)
Supplement: Additional file 3 — More results from Simulation I on type-I error and power. [file 12859_2014_439_MOESM3_ESM.pdf]

Supplementary Materials

Additional file 3: More results from Simulation I on type-I error and power

Supplementary Figure 5 summarizes the type-I error results from Simulation I, for $\tau = 0$ (shown in left column) and $\tau = 0.2$ (shown in right column), respectively. In the boxplots, each box represents the type-I errors from 1,000 simulations under a specific setting of m and δ. It can be seen that when fixing τ, changing δ does not have an obvious effect on type-I error in general. However for $m = 100$, increasing δ tends to results in larger type-I error. The reason is that when the number of reads is large, for larger δ, it is easier to form candidate groups therefore the chance of rejecting the null hypothesis tends to increase. Similarly, we draw boxplots for $\delta = 0.15$ (shown in left column) and $\delta = 0.35$ (shown in right column) in Supplementary Figure 6. From the figure we see a clear trend that when fixing δ, type-I error decreases as τ increases. In other words, larger τ corresponds to more conservative test.

For power analysis, we draw the power curves for different number of reads and different cell-type proportions under different settings of $\tau = 0, 0.2, 0.4$ and $\delta = 0.1, 0.2, 0.3, 0.4$ in Supplementary Figure 7. It can be seen that in general, power increases as the number of reads gets larger, also as the cell-type proportion becomes more balanced. In addition, when fixing τ, changing δ does not have an obvious effect on power in general; whereas when fixing δ, larger τ results in slightly reduced power since bipolar detection becomes more conservative.

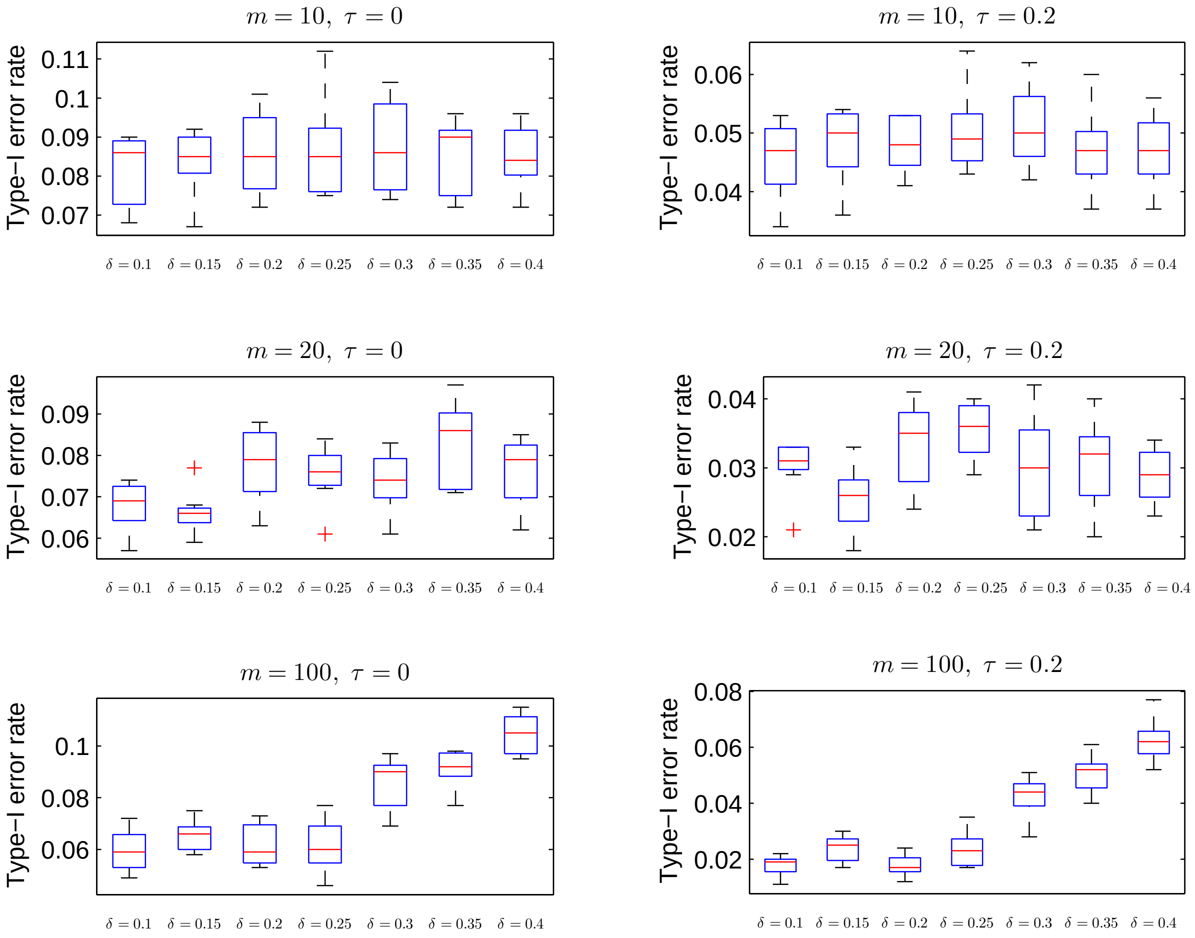

Supplementary Figure 5: Boxplot of type-I error in bipolar methylation detection, for different number of reads. Left column: when fixing $\tau = 0$ and setting different values for δ. Right column: when fixing $\tau = 0.2$ and setting different values for δ.

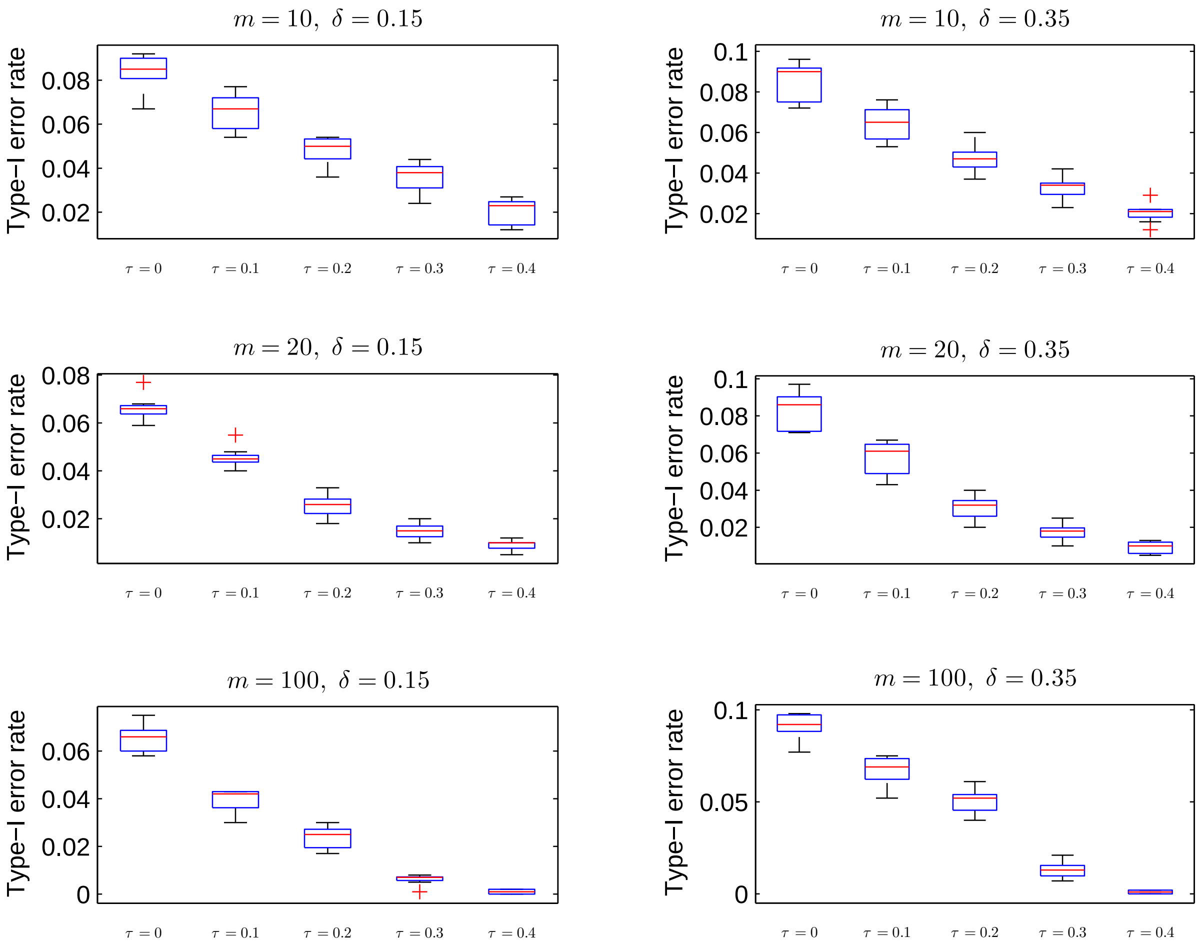

Supplementary Figure 6: Boxplot of type-I error in bipolar methylation detection, for different number of reads. Left column: when fixing $\delta = 0.15$ and setting different values for τ. Right column: when fixing $\delta = 0.35$ and setting different values for τ.

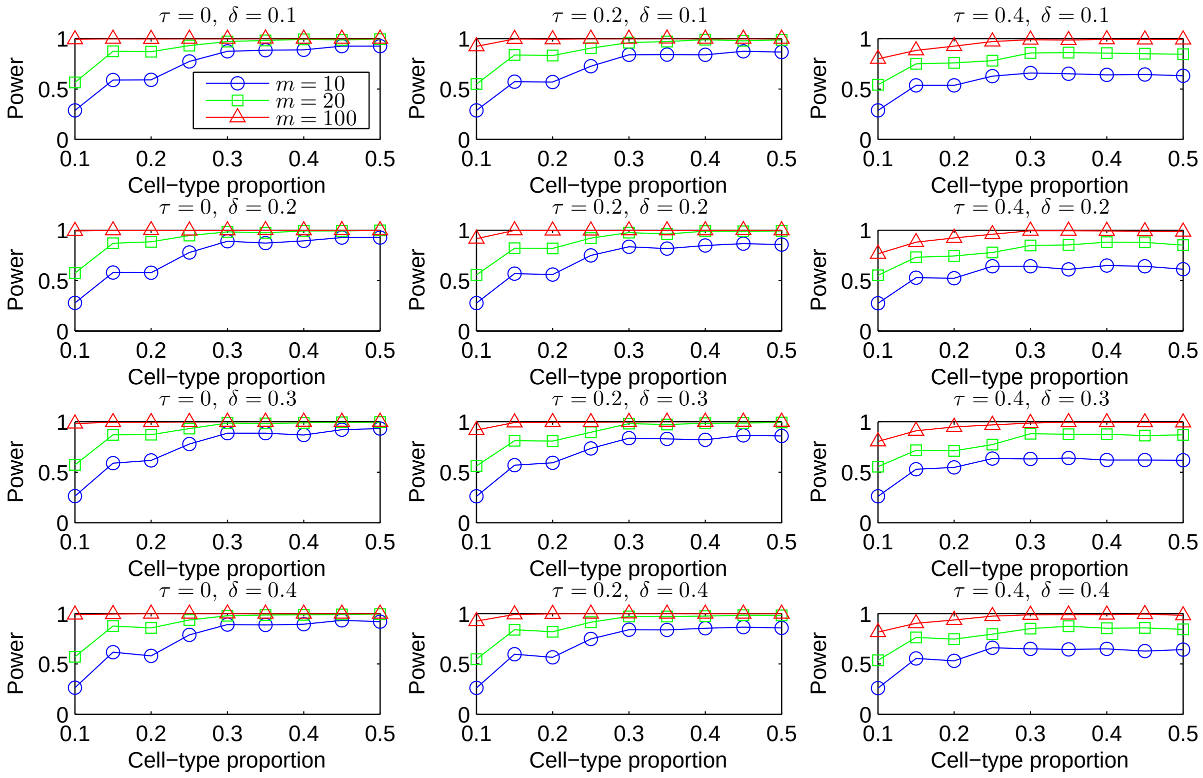

Supplementary Figure 7: Power in bipolar methylation detection, for different number of reads and different cell-type proportions when setting $\tau = 0, 0.2, 0.4$ and $\delta = 0.1, 0.2, 0.3, 0.4$.
